# The Effect of Annealing Ambience on the Material and Photodetector Characteristics of Sputtered ZnGa_2_O_4_ Films

**DOI:** 10.3390/nano11092316

**Published:** 2021-09-06

**Authors:** Anoop Kumar Singh, Shiau-Yuan Huang, Po-Wei Chen, Jung-Lung Chiang, Dong-Sing Wuu

**Affiliations:** 1 Department of Materials Science and Engineering, National Chung Hsing University, Taichung 40227, Taiwan; anoop.scns@gmail.com (A.K.S.); ashin120606@gmail.com (S.-Y.H.); iversonc110307@gmail.com (P.-W.C.); cjunglung@gmail.com (J.-L.C.); 2 Department of Applied Materials and Optoelectronic Engineering, National Chi Nan University, Nantou 54561, Taiwan; 3 Innovation and Development Center of Sustainable Agriculture, National Chung Hsing University, Taichung 40227, Taiwan

**Keywords:** ZnGa_2_O_4_, annealing ambience, wide bandgap, metal-semiconductor-metal photodetector, responsivity

## Abstract

Spinel ZnGa_2_O_4_ films were grown on c-plane sapphire substrates at the substrate temperature of 400 °C by radio-frequency magnetron sputtering. Post thermal annealing was employed at the annealing temperature of 700 °C in order to enhance their crystal quality. The effect of thermal annealing on the microstructural and optoelectronic properties of ZnGa_2_O_4_ films was systematically investigated in various ambiences, such as air, nitrogen, and oxygen. The X-ray diffraction patterns of annealed ZnGa_2_O_4_ films showed the crystalline structure to have (111) crystallographic planes. Transmission electron micrographs verified that ZnGa_2_O_4_ film annealed under air ambience possesses a quasi-single-crystalline structure. This ZnGa_2_O_4_ film annealed under air ambience exhibited a smooth surface, an excellent average transmittance above 82% in the visible region, and a wide bandgap of 5.05 eV. The oxygen vacancies under different annealing ambiences were revealed a substantial impact on the material and photodetector characteristics by X-ray photoelectron spectrum investigations. ZnGa_2_O_4_ film exhibits optimal performance as a metal-semiconductor-metal photodetector when annealed under air ambience. Under these conditions, ZnGa_2_O_4_ film exhibits a higher photo/dark current ratio of ~10^4^ order, as well as a high responsivity of 2.53 A/W at the bias of 5 V under an incident optical light of 240 nm. These results demonstrate that quasi-single-crystalline ZnGa_2_O_4_ films have significant potential in deep-ultraviolet applications.

## 1. Introduction

Deep-ultraviolet photodetectors (DUV PDs) based on ZnGa_2_O_4_ films are of substantial interest due to their prospective applications in flame monitoring, missile threat detection, ozone hole monitoring, engine control, optical communication, aerospace, and lithography alignment [1,2,3,4]. The high electrical conductivity and high electrochemical activities of ZnGa_2_O_4_ make it suitable for use as an anode material in rechargeable batteries [5]. ZnGa_2_O_4_ is a well-known phosphor material due to its intrinsic blue emission characteristics, which may be shifted to other emission wavelengths by doping metal ions or surface defects. It also exhibits a variety of functional features that can be applied to optoelectronic devices [6,7]. ZnGa_2_O_4_ possesses a wide bandgap in the region of 4.4–5.2 eV [8,9]. Its inherent chemical and thermal characteristics in a harsh environment along with high breakdown voltage make ZnGa_2_O_4_ a potential candidate for high-voltage devices, such as transistors and MOSFETs [10]. Wide bandgap semiconductors improve the efficiency of power-conversion stages, and they may be utilized instead of silicon in the production of voltage converters, power MOSFETs, and high-efficiency Schottky diodes. These can then be employed in electric and hybrid cars [11]. Recently, Chi et al. and Chikoidze et al. demonstrated the p-type ZnGa_2_O_4_ semiconductor (5 eV), which could pave the way for bipolar oxide energy electronics by reducing switching and conversion losses. This is due to ZnGa_2_O_4_′s combination of the required qualities for sustaining large electrical fields in p-n junctions in the off-state, together with low losses in the on-state [12,13]. ZnGa_2_O_4_ possesses the cubic symmetric spinel structure with the space group of Fd$\bar{3}$m, where Zn^2+^ cations occupy tetrahedral sites and Ga^3+^ cations occupy octahedral sites with oxygen atoms in close-packed cubic structures [11,14].

There are other wide bandgap semiconductors such as β-Ga_2_O_3_ and (Al_x_Ga_1−x_)_2_O_3_, but these require high growth temperatures as well as high post-thermal annealing temperatures in order to create high-quality epitaxial films to employ in DUV spectral selection. Besides this, its anisotropic structural, optical, thermal, and mechanical characteristics including easy cleaving are the other drawbacks associated with β-Ga_2_O_3_. These drawbacks can be overcome using spinel ZnGa_2_O_4_, which offers a higher electrical conductivity, an isotropic cubic structure, distinguishable Zn and Ga cation sites, and a stable phase [15]. ZnGa_2_O_4_ is found to be suitable for use as the active layer in the fabrication of DUV PDs due to its insensitivity to light above the wavelength of 280 nm. Optoelectronic devices fabricated with one-dimensional nanostructures, such as nanotubes, nanowires, and nanocrystals, can achieve a high photoelectric conversion efficiency. However, in practice their stability and repeatability are still major concerns [16,17,18]. Hence, the development of ZnGa_2_O_4_ film-based PDs is essential. ZnGa_2_O_4_ films have been reported earlier by several research groups using radio-frequency (RF) magnetron sputtering [19,20], pulsed laser deposition [21], metal-organic chemical vapor deposition (MOCVD) [22], and mist-CVD [23]. Among these, RF magnetron sputtering is a cost-effective and dependable technology that is extensively utilized by industrial processes to build commercial optoelectronic devices. This is due to its ability to deposit films with a high purity, excellent compactness, repeatability, and homogeneity across a large area in less time than that taken by other technologies.

In this study, RF magnetron sputtering is used for depositing ZnGa_2_O_4_ films. The microstructural and optoelectronic characteristics of ZnGa_2_O_4_ films have been systematically investigated using an X-ray diffractometer, transmission electron microscope, atomic force microscope, field-emission scanning electron microscope, N&K analyzer, Keithley semiconductor parameter analyzer, and deuterium lamp with an Omni spectrometer. To date, the ambient effects of these thermal annealing processes have not been explored when assessing the material and photodetector characteristics of ZnGa_2_O_4_ films on sapphire substrates using RF magnetron sputtering.

## 2. Materials and Methods

ZnGa_2_O_4_ films (200 nm thickness) were deposited on 2-inch c-plane sapphire (0001) substrates using RF magnetron sputtering (model: SP-203, LHUHV, Hsinchu, Taiwan). The 3-inch ceramic target of ZnGa_2_O_4_, which had a purity of 99.99% (with a 50/50 ratio of ZnO/Ga_2_O_3_), was taken into consideration. The substrate was cleaned with acetone, isopropyl alcohol, and distilled water separately. It was then blown-dried with nitrogen gas before being introduced into the deposition chamber. When the base pressure had reached 1 × 10^−6^ Torr, the deposition of films was performed at the working pressure of 5 × 10^−3^ Torr and the substrate temperature of 400 °C. The rotation of the sample stage was maintained at 10 rpm to achieve a uniform thickness. The flow rate of argon gas was maintained at 10 sccm. Prior to sputtering, the target was pre-sputtered for 10 min by covering the target of the shutter in order to remove impurities on the surface of the ZnGa_2_O_4_. After this, the plasma was ignited by directing 100 W of RF power on the ceramic ZnGa_2_O_4_ target. As-deposited films were thermally annealed at a temperature of 700 °C using rapid thermal annealing (RTA, RTP-T41, Premtek International Inc., Hsinchu, Taiwan) for 1 min under air, nitrogen, and oxygen ambiences.

The thickness of the films was determined by an α-step profile analyzer. The crystallinity of the films was measured using a X-ray diffractometer (HR-XRD, X’Pert Pro MRD, PANanalytical, Almelo, Netherland). An N&K analyzer (model: 1280, n&k Technologies, San Jose, CA, USA) was used in this investigation to measure the transmittance of the ZnGa_2_O_4_ films. The optical bandgap of the films was extracted through Tauc plot. The surface morphology was determined using a field emission scanning electron microscope (FESEM, JSM-6700F, JEOL, Tokyo, Japan). The root-mean-square (RMS) surface roughness of the ZnGa_2_O_4_ films was measured using an atomic force microscope (AFM, Dimension 5000, Bruker, Santa Clara, CA, USA). The nanostructure and crystal orientations of the ZnGa_2_O_4_ films were studied using a transmission electron microscope (JEM-2100F, JEOL, Tokyo, Japan). The chemical states of the films were characterized by X-ray photoelectron spectroscopy (XPS, PHI 5000 Versa Probe, ULVAC-PHI, Kanagawa, Japan). The X-ray source for XPS was the monochromatized AlKα source (1486 eV). The X-ray beam size and takeoff angle were 100 µm and 45°, respectively. The pass energy was 58.7 eV. High-resolution scans were obtained by averaging 50 scans for O 1s peak in this investigation. Surface charging was minimized by an electron flood gun operated at 3 eV.

The metal-semiconductor-metal photodetector was fabricated with the interdigitated metal Ti/Au (with a thickness of 40/60 nm) Schottky contacts with the help of an electron-beam evaporator. These contacts were specified by photolithography and lift-off techniques. The active areas of the PDs were 1.05 × 1.05 mm^2^, whereas the length, width, and spacing for the interdigital electrodes were specified as 950, 50, and 50 μm, respectively. The current–voltage characteristics were measured via a Keysight/Agilent semiconductor parameter analyzer (4155B, Hewlett-Packard Company, Englewood, CO, USA). A 30 W deuterium lamp was associated with the spectrometer (Omni-λ3029i, Zolix, Beijing, China) as the light source in the wavelength region of 200–360 nm, which helped us in the measurement of the spectral response of ZnGa_2_O_4_ PDs at the bias of 5 V.

## 3. Results and Discussion

**Figure 1:**
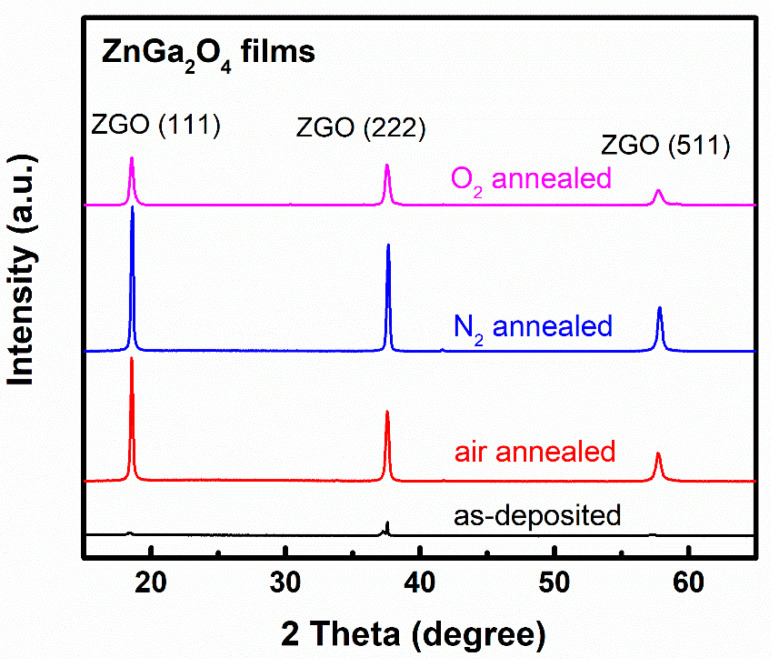
X-ray diffraction patterns of ZnGa_2_O_4_ (ZGO) films under different annealing ambiences.

Figure 1 shows the X-ray diffraction patterns of as-deposited and annealed ZnGa_2_O_4_ films under different annealing ambiences. As-deposited ZnGa_2_O_4_ film exhibited weak Bragg reflections for the (111), (222), and (511) planes, which corresponds to the spinel-cubic ZnGa_2_O_4_ (JCPDS card no-381240). The crystallinity of the as-deposited ZnGa_2_O_4_ film improved after thermal annealing under different ambiences. The thermal annealing of ZnGa_2_O_4_ films under air and nitrogen ambience dramatically increased the intensity of the (111), (222), and (511) planes, indicating that these films had a high crystallinity. In contrast, ZnGa_2_O_4_ film annealed under oxygen ambience had a low crystallinity, implying that this as-deposited film became oxidized after thermal annealing under an oxygen atmosphere. This shows that annealing in oxygen ambience can fulfill the oxygen vacancies in the ZnGa_2_O_4_ film, which leads to its oxidation and thus slight degradation in its crystallinity.

**Figure 2:**
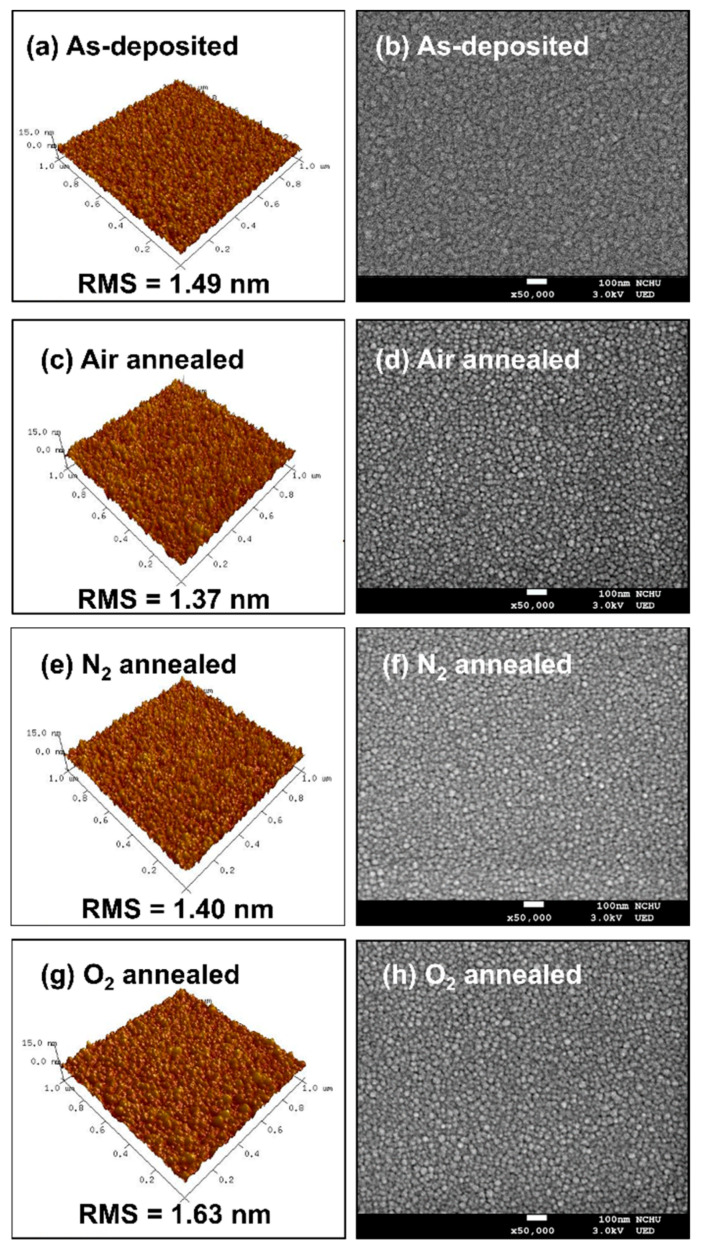
AFM micrographs of (**a**) as-deposited, (**c**) air annealed, (**e**) N_2_ annealed, and (**g**) O_2_ annealed ZnGa_2_O_4_ films; SEM micrographs of (**b**) as-deposited, (**d**) air annealed, (**f**) N_2_ annealed, and (**h**) O_2_ annealed ZnGa_2_O_4_ films.

Figure 2a–h shows the AFM and SEM micrographs of as-deposited and annealed ZnGa_2_O_4_ films under different annealing ambiences. The RMS surface roughness of the as-deposited ZnGa_2_O_4_ film was 1.49 nm, whereas the RMS surface roughness of the annealed ZnGa_2_O_4_ film under air, nitrogen, and oxygen ambience was 1.37, 1.40, and 1.63 nm, respectively. This indicates that the ZnGa_2_O_4_ film annealed under air and nitrogen ambience had a smooth surface, which is beneficial for PDs. A smooth surface leads to a lower surface area, which results in low surface density states for the ZnGa_2_O_4_ film. The RMS surface roughness was found to be relatively larger after annealing under oxygen ambience, which could be due to the large amount of oxygen diffused by the adsorption of oxygen in the ZnGa_2_O_4_ film [24]. The SEM micrographs of the as-deposited ZnGa_2_O_4_ film revealed the columnar structure and irregular arrangement of grains with a rough surface, which were found to be dense and smooth after annealing under air and nitrogen ambiences with the presence of some nano-voids. It is well-known that high-temperature annealing reduces the surface energy between grains, which increases grain growth and reduces surface roughness due to the coalescence of small grains [25,26]. ZnGa_2_O_4_ film annealed under oxygen ambience was found to be oxidized due to the absorption of oxygen in the film, which possesses a dense and highly rough surface with fewer nano-voids than other films [24]. These SEM results were found to be in accordance with the AFM and XRD results.

**Figure 3:**
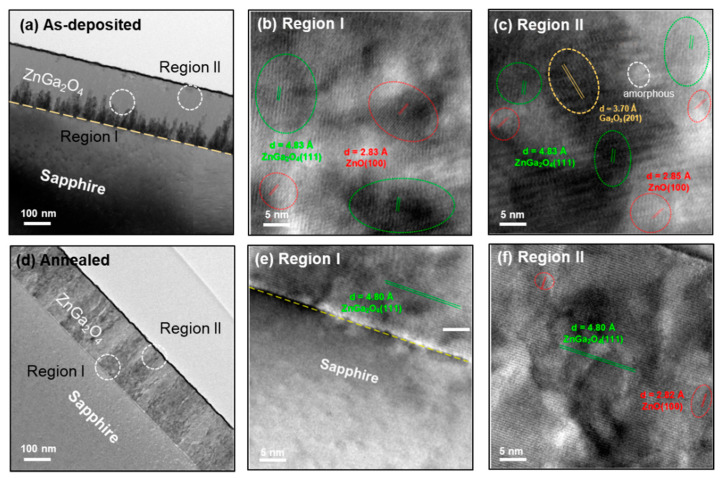
Cross-sectional and HRTEM micrographs of (**a**) as-deposited, (**b**) region I, (**c**) region II of as-deposited ZnGa_2_O_4_ film, (**d**) annealed, (**e**) region I, and (**f**) region II of annealed ZnGa_2_O_4_ film under air ambience.

Figure 3 depicts the cross-sectional and high-resolution transmission electron micrographs (HRTEM) of as-deposited and annealed ZnGa_2_O_4_ film under air ambience. The cross-sectional TEM micrograph of as-deposited ZnGa_2_O_4_ film is shown in Figure 3a, while region I and region II of this film are explored in Figure 3b,c, respectively. This as-deposited ZnGa_2_O_4_ film demonstrated columnar grains near the substrate region and possessed the amorphous, ZnO (100), Ga_2_O_3_ (201), and ZnGa_2_O_4_ (111) phases. These phases are evident in this film, as it was deposited at a substrate temperature of 400 °C. The d-spacing of ~2.83, 3.70, and 4.83 Å (compatible with 4.80 Å) was measured, which corresponded to the ZnO (111), Ga_2_O_3_(201), and ZnGa_2_O_4_ (111) phases, respectively. The rapid thermal annealing was performed at the temperature of 700 ℃ for 1 min under air ambience to improve the crystallinity of this film. The oxygen present in the air ambience during thermal annealing played a significant role in eliminating the intrinsic defects, such as amorphous regions and oxygen vacancies in the films. A cross-sectional TEM micrograph of the annealed ZnGa_2_O_4_ film under air ambience is shown in Figure 3d, and its region I and region II are explored in Figure 3e,f, respectively. These regions (I and II) of the annealed ZnGa_2_O_4_ film demonstrated that rapid thermal annealing under air ambience suppressed the amorphous and Ga_2_O_3_ phases by the local bond rearrangement at the interface between the amorphous phase and the crystalline phase, and achieved a quasi-single-crystalline ZnGa_2_O_4_ structure, as shown in Figure 3e,f.

**Figure 4:**
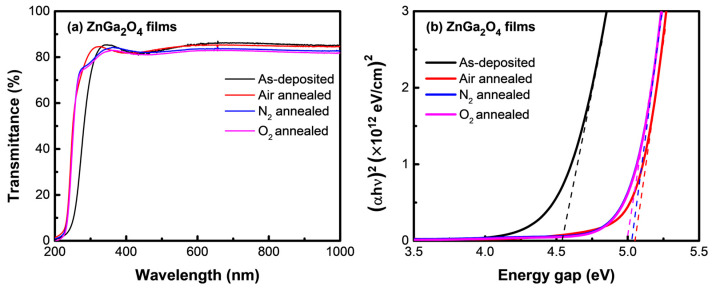
(**a**) Transmittance spectrum and (**b**) Tauc plot of the as-deposited and annealed ZnGa_2_O_4_ films under different annealing ambiences.

Figure 4a shows the transmittance spectrum of the as-deposited and annealed ZnGa_2_O_4_ films under different annealing ambiences. The average transmittance of the as-deposited and annealed ZnGa_2_O_4_ films was over 82% in the visible range, with a sharp cut-off in the DUV region. The absorption band-edge of the as-deposited film shifted to shorter wavelengths upon thermal annealing, which demonstrated the improvement in the optical characteristics of this film. Figure 4b shows the Tauc plot, which demonstrates the bandgap values for the as-deposited and annealed ZnGa_2_O_4_ films under different ambiences. The transmittances values of these films were taken into consideration to calculate the absorption coefficient α. The optical bandgap of ZnGa_2_O_4_ films was obtained using the following relation:

(αhν) = A (hν − E_g_)^1/2^ (1)

where A, α, hν, and E_g_ represent the constant, absorption coefficient, energy of the incident photon, and the bandgap energy, respectively. The narrow bandgap of 4.53 eV in the as-deposited ZnGa_2_O_4_ film can be attributed to the existence of defect states (amorphous structure, phase separations between Ga_2_O_3_ and ZnO), as shown by TEM (see Figure 3b,c). This led the excited electron to migrate to the conduction band with a low photon energy, narrowing the bandgap. Thermal annealing in various ambiences mitigates the defects (including amorphous nature and oxygen vacancies) that are common in as-deposited ZnGa_2_O_4_ film. As the thermal annealing was employed, the bandgap increased as 4.98, 5.02, and 5.05 eV under oxygen, nitrogen, and air ambiences, respectively, which allowed this material to lie within the DUV region. It is widely known that materials with a bandgap greater than 4.4 eV absorb UV-C radiation while ignoring visible light, making them ideal for use as high-sensitivity DUV PDs.

**Figure 5:**
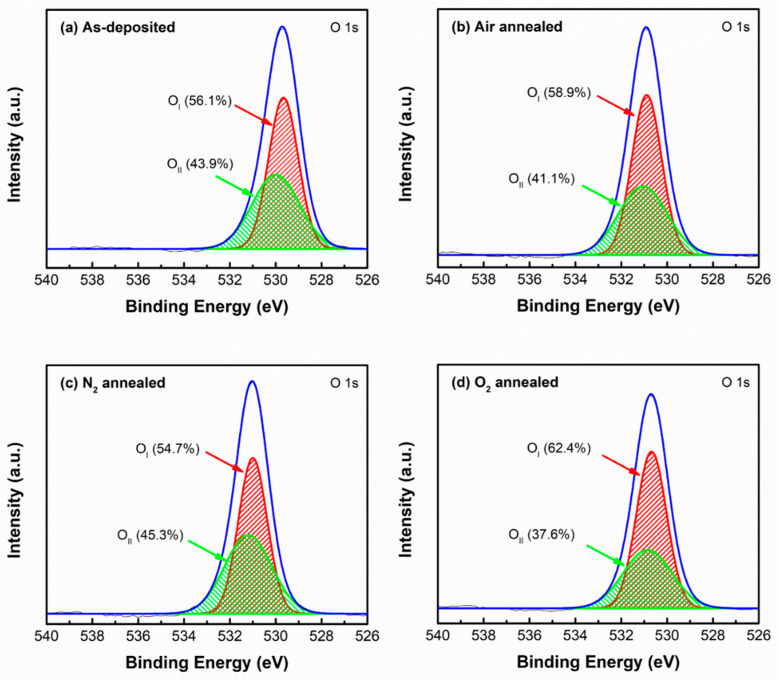
O 1s XPS spectra of (**a**) as-deposited, (**b**) air annealed, (**c**) N_2_ annealed, and (**d**) O_2_ annealed ZnGa_2_O_4_ films.

Figure 5 shows the O 1s XPS spectra of the as-deposited and annealed ZnGa_2_O_4_ films under different ambiences. In order to define the oxygen vacancies associated with the films, the O 1s XPS spectra of the films were fitted with the Gaussian function and deconvoluted into two peaks named O_I_ and O_II_. The O_I_ peak centered around the lower binding energies of 530–531 eV represents the oxygen deficiency in the films, whereas the O_II_ peak centered at higher binding energies of 531–532 eV represents the oxygen vacancies in the film [27]. The integrated areas of the O_II_ peak of the ZnGa_2_O_4_ films were found to be 43.9, 41.1, 45.3, and 37.6% for as-deposited, air annealed, N_2_ annealed, and O_2_ annealed ambiences, respectively. The oxygen present in the air ambience can fill a certain amount of oxygen vacancy. Hence, the area of O_II_ peak was found to be decreased from 43.9 to 41.1% during air annealing, as shown in Figure 5b. The area of the O_II_ peak was found to be higher (45.3%) in an N_2_ atmosphere, which can be attributed to the disassociation of oxygen molecules from the films during annealing. This resulted in an increased number of oxygen vacancies, as shown in Figure 5c [25]. By observing the integrated areas of O_II_ peaks from Figure 5a–d, it was found that the O_2_-annealed ZnGa_2_O_4_ film had the lowest integrated area of O_II_ peaks (37.6%). It is well-known that an oxygen-rich ambience reduces the number of oxygen vacancies, which weakens the crystalline structure [28]. This phenomenon is in accordance with our XRD results, as shown in Figure 1, where oxygen ambience deteriorated the crystalline structure. Since oxygen vacancies affect the optoelectronic properties of ZnGa_2_O_4_ film, the considerable suppression of oxygen vacancies demonstrates the tunability of the film characteristics depending on the annealing ambiences.

**Figure 6:**
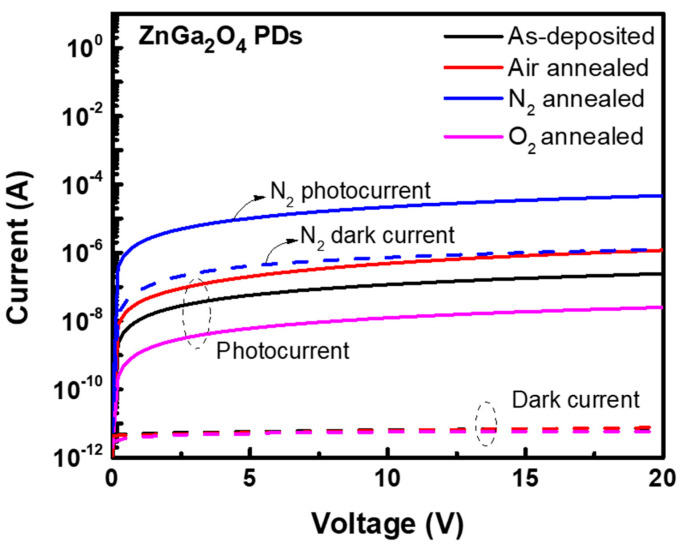
I–V characteristics of the as-deposited and annealed ZnGa_2_O_4_ PDs under different annealing ambiences.

Figure 6 shows the I–V characteristics of the as-deposited and annealed ZnGa_2_O_4_ PDs under different annealing ambiences. The as-deposited ZnGa_2_O_4_ PD exhibited the photocurrent and dark current of 5.69 × 10^−8^ A and 5.77 × 10^−12^ A, respectively. This photocurrent had decreased from 5.69 × 10^−8^ to 6.12 × 10^−9^ A after annealing under oxygen ambience. This decreased photocurrent can be ascribed to the fulfilment of oxygen vacancies during high temperature annealing under oxygen ambience, which oxidized the ZnGa_2_O_4_ films and resulted in the decreased value of photocurrent. Nitrogen ambience caused the desorption of oxygen atoms from the surface of the films, and created oxygen vacancies, which drastically increased the photocurrent of the as-deposited ZnGa_2_O_4_ PD from 5.69 × 10^−8^ to 9.64 × 10^−6^ A. The dark current of as-deposited ZnGa_2_O_4_ PD was also found to be increased from 5.77 × 10^−12^ to 3.33 × 10^−7^ A upon annealing this film under nitrogen ambience, which was not desirable for PDs. This high dark current value can be attributed to the high number of inherent defects in the ZnGa_2_O_4_ PDs, such as oxygen vacancies and high surface density states, which can result in trapping states and was responsible for a higher internal gain. As a result, these surface states enhanced the dark current as well as the nonradiative recombination for the ZnGa_2_O_4_ film (annealed under nitrogen ambience). Similar results have been observed by Tsai et al. in their work [29], where surface related defects were the main cause of the large leakage current in their device, which acted as the adsorption site and captured the free electrons in ZnGa_2_O_4_ films. When air ambience was used for annealing, ZnGa_2_O_4_ PD exhibited a photocurrent of 2.02 × 10^−7^ A, a dark current of 5.35 × 10^−12^ A, and a I_ph_/I_dark_ of 3.77 × 10^4^. Annealing ZnGa_2_O_4_ film under air ambience can fill a significant proportion of oxygen vacancies from the oxygen present in the air ambience, which exhibits the enhancement in optoelectronic characteristics of this ZnGa_2_O_4_ film and its potential to employ in DUV PDs.

**Figure 7:**
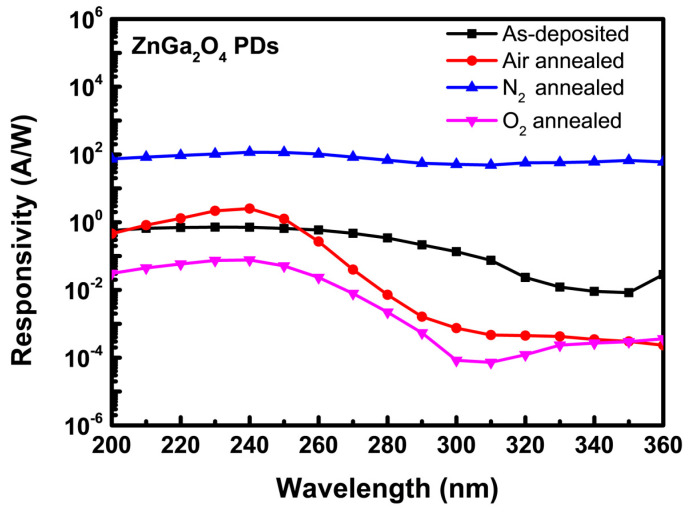
Spectral response of as-deposited and annealed ZnGa_2_O_4_ PDs under different annealing ambiences.

Figure 7 shows the spectral response of the as-deposited and annealed ZnGa_2_O_4_ PDs under different annealing ambiences. The spectral response describes the sensitivity of the photodetector to optical radiation of various wavelengths, which is known as the responsivity of the photodetector. This parameter defines the performance of the photodetector and is represented by the following relation:

R = (I_ph_-I_dark_)/P_inc_ (2)

where I_ph_ is the photocurrent in Ampere (A), I_dark_ is the dark current in Ampere (A), and P_inc_ is the incident optical power in Watt (W). Hence, the responsivity of the photodetector is measured in A/W. As-deposited ZnGa_2_O_4_ PD possessed a responsivity of 0.71 A/W, which decreased to 0.12 A/W upon thermal annealing under oxygen ambience. This decreased responsivity could be due to the fulfilment of oxygen vacancies by oxygen gas, which further decreased the mobility of carriers and exhibited the poor responsivity of the ZnGa_2_O_4_ PD. Annealing under nitrogen ambience drastically increased the responsivity of ZnGa_2_O_4_ PD from 0.71 to 117 A/W. However, N_2_ annealed ZnGa_2_O_4_ PD exhibited a broad spectral response from the DUV region to the visible region, which was not suitable for the DUV PDs. Annealing under nitrogen ambience reduced the oxygen concentration from the film and generated oxygen vacancy defects and surface density states. These defects led to the creation of deep-trapping centers, which increased the photocurrent as well as the dark current and enhanced the internal gain for ZnGa_2_O_4_ PD [29]. Zheng et al. and Han et al. reported that the high responsivity of Mg_0.46_Zn_0.54_O and Mg_0.52_Zn_0.48_O films-based photodetectors is due to the long lifetime of photo-ionized holes, which were found to be trapped at the deep level. This measurement was precisely taken by using deep-level spectral measurement system in their works [30,31]. The trap states of holes include interface states between semiconductor and electrode, surface states, and deep level defects. These trap states were found responsible for this long lifetime phenomenon. It is clear that the photoresponse characteristics of ZnGa_2_O_4_ PDs could represent variations due to various oxygen vacancy related defects, which can be suppressed by the adaptation of air ambience. A large amount of oxygen molecules or atoms in the air ambience will diffuse into the samples and fill the significant number of oxygen vacancies, resulting in low surface density states [32]. Annealing under air ambience exhibited both a significant enhancement in the photocurrent and reduction in the dark current by providing adequate energy to compensate the oxygen vacancy related defects. Hence, the responsivity of 2.53 A/W at the given bias of 5 V under the incident optical light of 240 nm was obtained for ZnGa_2_O_4_ PD under air ambience. The performance of our fabricated device is compared with that of the previously reported ZnGa_2_O_4_ PDs in Table 1.

**Figure 8:**
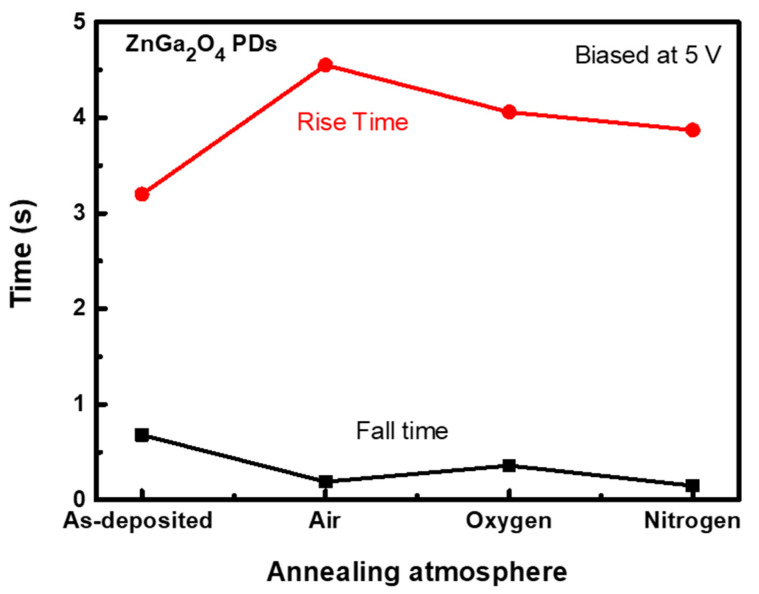
Time response of as-deposited and annealed ZnGa_2_O_4_ PDs under different annealing ambiences.

Figure 8 shows the rise time and fall time for the as-deposited and annealed ZnGa_2_O_4_ PDs under different annealing ambiences. Rise time is the time that the PD takes to change its response from 10% to 90% of the maximum photocurrent with the illumination of light. Fall time is the time that the PD takes to change its response from 90% to 10% of the maximum photocurrent without the illumination of light. These two characteristics (rise time and fall time) represent the response time of a photodetector. ZnGa_2_O_4_ PD annealed under air ambience possesses a higher rise time of 4.55 s but a lesser fall time of 0.19 s than other PDs (as-deposited, annealed ZnGa_2_O_4_ PDs under nitrogen and oxygen ambiences), which could be due to the indirect bandgap properties of the spinel-cubic ZnGa_2_O_4_ structure [36]. The high rise time for the ZnGa_2_O_4_ PD annealed under air ambience could be attributed to the excitation of photogenerated carriers, which reached higher energy states above the conduction band. These excited photogenerated carriers then reverted to the conduction band by releasing energy and resulted in a high rise time. Despite the fact that the response time of ZnGa_2_O_4_ PDs annealed in air ambience is higher than that of other ZnGa_2_O_4_ PDs annealed under other ambiences, the responsivity associated with this air annealed ZnGa_2_O_4_ PD is found to be considerable for use in DUV PDs.

## 4. Conclusions

We have successfully deposited ZnGa_2_O_4_ films on sapphire substrates using RF magnetron sputtering, as well as studied the effects of thermal annealing in various ambiences (such as air, nitrogen, and oxygen) on the material and photodetector characteristics of ZnGa_2_O_4_ films. Investigations using XRD, AFM, SEM, TEM, N&K analyzer, and DUV measurements revealed that the microstructural and optoelectronic characteristics of ZnGa_2_O_4_ films were enhanced under air ambience and affected under nitrogen and oxygen ambiences. These results were verified by comparatively analyzing the chemical states of oxygen on the surface of the ZnGa_2_O_4_ films before and after annealing in different ambiences using XPS. The X-ray diffraction patterns of the annealed ZnGa_2_O_4_ films exhibited crystalline structures with (111) crystallographic planes. The as-deposited ZnGa_2_O_4_ film possessed amorphous regions and phase separations between Ga_2_O_3_ and ZnO, which were found to be suppressed after annealing under air ambience. The TEM micrographs revealed that ZnGa_2_O_4_ film annealed under air ambience possessed a quasi-single-crystalline structure. The average transmittance of 82% in the visible region and wide bandgap of 5.05 eV were observed for this ZnGa_2_O_4_ film. The metal-semiconductor-metal PD fabricated by employing this air annealed ZnGa_2_O_4_ film exhibited the high responsivity of 2.53 A/W at the bias of 5 V under the incident light of 240 nm. Besides this, ZnGa_2_O_4_ PD had a rise time of 4.55 s, a fall time of 0.19 s, and a photo/dark current ratio of ~10^4^ order. These results indicate that quasi-single-crystalline ZnGa_2_O_4_ films have a high potential to employ in DUV PDs.

## Figures and Tables

**Table 1:** Comparison of some key performance of our ZnGa_2_O_4_ PD with other previously reported ZnGa_2_O_4_ PDs.

Material	Growth Method	I_dark_ (A)	I_ph_ (A)	R (A/W)	Bias Voltage and λ_peak_	Reference
ZnGa_2_O_4_	MOCVD	~10^−14^	2.93 × 10^−6^	0.203	10 V, 260 nm	[33]
ZnGa_2_O_4_	MOCVD	0.86 × 10^−12^	4.04 × 10^−8^	0.46	10 V, 230 nm	[34]
ZnGa_2_O_4_	Sputter	2.70 × 10^−11^	-	-	2 V, 255 nm	[35]
ZnGa_2_O_4_	Sputter	5.35 × 10^−12^	2.02 × 10^−7^	2.53	5 V, 240 nm	This work

## Data Availability

The data that support the findings of this study are available from the corresponding author upon reasonable request.
